# Physiological Mechanisms Inherent to Diabetes Involved in the Development of Dementia: Alzheimer’s Disease

**DOI:** 10.3390/neurolint15040079

**Published:** 2023-10-10

**Authors:** Himan Mohamed-Mohamed, Victoria García-Morales, Encarnación María Sánchez Lara, Anabel González-Acedo, Teresa Pardo-Moreno, María Isabel Tovar-Gálvez, Lucía Melguizo-Rodríguez, Juan José Ramos-Rodríguez

**Affiliations:** 1 Department of Physiology, Faculty of Health Sciences of Ceuta, University of Granada, 51001 Ceuta, Spain; 2 Physiology Area, Department of Biomedicine, Biotechnology and Public Health, Faculty of Medicine, University of Cádiz, Pl. Falla, 9, 11003 Cádiz, Spain; 3 Department of Personalidad, Evaluación y Tratamiento Psicológico, Faculty of Health Sciences (Ceuta), University of Granada, 51001 Ceuta, Spain; emsanchez@ugr.es; 4 Department of Nursing, Faculty of Health Sciences of Ceuta, University of Granada, 51001 Ceuta, Spain; 5 Biomedical Group (BIO277), Department of Nursing, Faculty of Health Sciences, University of Granada, 18016 Granada, Spain

**Keywords:** Alzheimer’s disease, dementia, vascular dementia, diabetes, insulin, neurodegeneration, neuron death, neuroinflammation

## Abstract

Type 2 diabetes mellitus (T2D) is a metabolic disease reaching pandemic levels worldwide. In parallel, Alzheimer’s disease (AD) and vascular dementia (VaD) are the two leading causes of dementia in an increasingly long-living Western society. Numerous epidemiological studies support the role of T2D as a risk factor for the development of dementia. However, few basic science studies have focused on the possible mechanisms involved in this relationship. On the other hand, this review of the literature also aims to explore the relationship between T2D, AD and VaD. The data found show that there are several alterations in the central nervous system that may be promoting the development of T2D. In addition, there are some mechanisms by which T2D may contribute to the development of neurodegenerative diseases such as AD or VaD.

## 1. Introduction

Diabetes mellitus (DM) is a chronic disease characterized by elevated blood glucose levels due to the body’s inability to produce sufficient insulin or use it efficiently. Although there are different types of DM, the most common are type 1 diabetes (T1D) and type 2 diabetes (T2D) [1].

**Figure 1:**
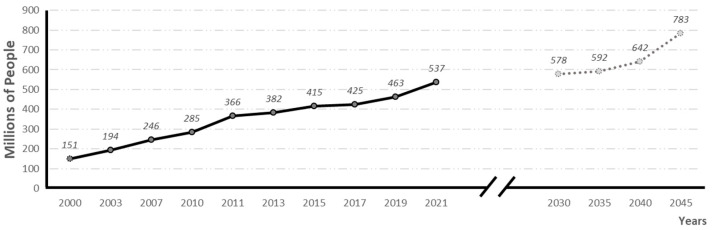
Global prevalence of diabetes and global prevalence predictions (adapted from 10th edition of IFD diabetes Atlas) [2].

According to the International Diabetes Federation (IDF, 2021), diabetes is the fifth leading cause of death in the world. In 2021, 537 million people were suffering from the disease, and it is estimated that by 2030, 783 million people will be affected (see evolution and prevalence forecasts in Figure 1). In 2021 alone, 6.7 million people died from diabetes, 1 every 5 s [2].

On the other hand, dementia is a syndrome that encompasses a heterogeneous group of diseases of the central nervous system (CNS), characterized by cognitive impairment [3]. In terms of incidence, the main types of dementia are Alzheimer’s disease (AD), vascular dementia (VaD), dementia with Lewy bodies and frontotemporal lobar dementia [4]. According to a WHO report, dementia currently affects around 50 million people, mostly from low- and middle-income countries, and about 10 million cases are registered every year [5]. It is estimated that by 2030, 82 million people will have dementia [5], and this number will increase to 130 million by 2050 [6].

AD is a neurodegenerative disease of the central nervous system and is characterized by a progressive deterioration of higher brain functions, affecting the ability to make decisions and execute them [7,8]. AD patients survive an average of 7 years, and slightly less than 3% of those affected live more than 14 years after diagnosis [9]. In terms of epidemiology, AD is the leading cause of dementia and occurs most frequently in people over 65 years of age (with a 10% prevalence), representing between 60% and 75% of all dementia cases [10,11]. The major risk factor for AD is age, and as life expectancy progressively increases, so does the number of people affected by this disease. Currently, more than 50 million people worldwide suffer from AD, with an associated economic cost of 30,000 EUR/year/patient in developed countries [12]. Projections for 2050 suggest that the number of people affected could exceed 107 million [10]. These rates represent an unbearable economic and social drain.

After AD, VaD is the second most common cause of cognitive impairment (20–30%) affecting 1 in 20 people over 65 years of age [3,13,14]. However, both diseases can coexist [15]. VaD incidence does not follow a homogeneous pattern geographically. Thus, in North America and Europe, it accounts for 15–20% of all dementias, while in Asia and developing countries, it is 30% [14]. As with all other dementias, the main risk factor is age, with the probability of developing dementia doubling every 5.3 years [16]. VaD occurs due to reduced blood flow to the brain, causing damage to different brain structures. This decrease may be caused by cerebrovascular accidents or diseases affecting the blood vessels of the brain, such as arteriosclerosis. Impairment of cognitive functions may vary depending on the location and extent of brain damage caused by reduced blood flow. Although impairment of cognitive functions may vary depending on the location and extent of brain damage, the most common include processing speed, attention, memory, language and communication, as well as executive functioning [17]. Prevention and control of vascular risk factors, such as high blood pressure, DM and high cholesterol, can help reduce the risk of VaD [15].

### 1.1. Historical Development of Diabetes Mellitus

Society has been affected by DM since ancient times. The first record of its existence dates back to the 15th century AC when the disease was described in Ebers papyrus, found in Egypt. In the 2nd century AC, Aretaeus gave the name diabetes (Greek for “to pass through”) to this ailment because of the polyuria suffered by the patients. But it was not until 1679 that Thomas Willis made a masterly description of the disease, attributing the second name of mellitus (honey flavor) to the disease, given the sweet taste of the urine of sufferers. In the early 19th century, the French clinician Bouchardat associated DM with obesity and a sedentary lifestyle. Paul Langerhans gave a major impetus to basic diabetes research in 1869, when he published his doctoral thesis on the histology of the pancreas where he described the pancreatic beta cells, which formed isolated islets in the pancreas, to which he gave his name, and attributed the ability to synthesize the hormone responsible for the regulation of blood sugar levels. Shortly afterward, in 1889, the surgeons Von Mering and Minkowsky observed that, after removing the pancreas from animals, they became diabetic. All the evidence suggested that the pancreas produced a substance that was released into the blood, the absence of which was responsible for diabetes. This substance was not found or described until 1921 by Frederick Grant Banting and John James Richard Macleod, who succeeded in isolating insulin. They discovered that insulin is produced in the pancreas, in beta cells located in the islets of Langerhans, and demonstrated its hypoglycemic effect. A year later, they described the beneficial effect of treating diabetic patients with pancreatic extracts [18] and received the Novel Prize in Medicine in 1923 for their discoveries. Pig pancreatic extracts were used to treat diabetes until recombinant human insulin was commercialized in 1982.

### 1.2. Pathophysiological Characteristics of Diabetes Mellitus

As mentioned above, DM is broadly subdivided into two main types: T1D and T2D, with the latter accounting for 90% of all diabetes cases [19]. Although there is a deficiency of insulin production by pancreatic beta cells in both forms, the two types have different histopathological characteristics.

Thus, T1D is an autoimmune disease in which up to 99% of pancreatic islets are eliminated [20], with a consequent lack of insulin leading to a metabolic decompensation called ketoacidosis. T1D usually develops at an early age, while T2D usually occurs from the age of 40 years onward. However, due to the increasing prevalence of obesity, T2D has also been observed in younger people [21,22,23]. The last form of the disease also has insufficient insulin production, similar to T1D, although to a lower magnitude. Thus, the loss of beta mass in T2D is estimated to be approximately 40% (25–60%) [24], whereas in T1D, this loss may affect 90% of beta cells.

**Figure 2:**
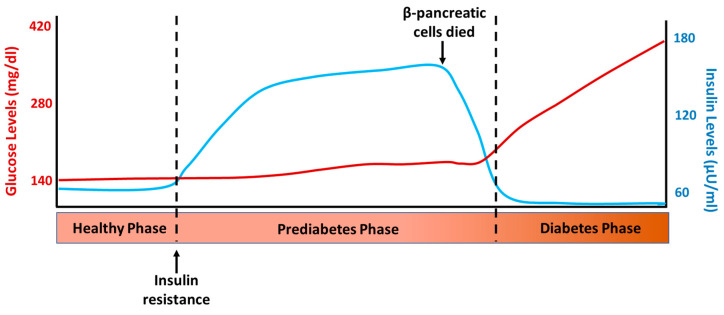
Representative levels of glucose and insulin after 1 h intake in the different phases of type 2 diabetes development.

T2D is a chronic disease characterized by defects in insulin action (insulin resistance) and secretion [25]. As a result, glucose accumulates in the bloodstream instead of entering the cells, leading to elevated blood sugar levels [2]. The sequence of events that occur in the development of T2D can be observed in Figure 2, which depicts the normal insulin and glucose levels one hour after a meal at the different stages of this disease. The first event in the sequence of processes leading to T2D is insulin resistance, which leads to increased insulin synthesis and secretion and compensatory hyperinsulinemia. This allows metabolic homeostasis to be maintained in the early stages of the disease; this compensatory insulin phase is called prediabetes and can last for years [26]. Once the balance between insulin resistance and secretion is disrupted, biochemical expression (glucose intolerance) and subsequent clinical diabetes begin. Patients with impaired glucose tolerance and short-standing diabetics have hyperinsulinemia, which is a common component of insulin resistance or metabolic syndrome. The loss of β-cells in DM implies that insulin secretion could be restored and hyperglycemia (but not hyperinsulinemia) normalized through the replacement or regeneration of the islets of Langerhans [27]. In fact, it has long been known that hyperglycemia in T1D and T2D can be reversed by pancreas transplantation [28] and the transplantation of isolated islets [29]. However, the number of transplantable pancreases is insufficient for the ever-increasing number of patients with DM, which impedes the widespread application of this intervention and promotes the continued search for other possible therapies for the treatment of this disease.

Other components of this syndrome that are related to insulin resistance and/or hyperinsulinemia are hypertension, dyslipidemia, obesity, gout, increased prothrombotic factors, fibrinolysis defects, atherosclerosis and dementia. Thus, individuals with T2D are at an increased risk of developing cardiovascular disease. These risks have been extensively studied in clinical and epidemiological research [30,31,32,33,34,35] as well as in basic research [36,37,38,39,40].

### 1.3. Historical Development of Dementias: Alzheimer’s Disease

The first historical allusion to the term dementia is found in the poem “De rerum natura” by Titus Lucretius in the 1st century BC and in the essay “De Senectute” by Cicero in which dementia is presented as a memory loss contracted with age. In this era, the average life expectancy was around 30 years, so it can be inferred that the term dementia encompasses other pathologies that affect psychiatric disorders. Etiologically, the word dementia means “absent mind” [41]. For centuries, the word dementia has encompassed a heterogeneous set of pathologies that present with memory loss and/or cognitive impairment. It was not until the 17th century that the definitions of dementia and cognitive decline became more precise, distinguishing between mental retardation and age-related cognitive impairment syndromes [41]. By the 19th century, the concept of dementia had acquired the current meaning found in medical literature. The Diagnostic and Statistical Manual of Mental Disorders defines dementia as a syndrome that includes memory loss and the loss of cognitive functions, which incapacitate those who suffer from it to lead a normal work, social and family life [42].

In 1906, German scientist Alois Alzheimer published the results of a study on Auguste Deter, a 52-year-old patient whose husband had brought her to the hospital after detecting changes in her behavior consistent with dementia. This study describes neurofibrillary tangles and senile plaques as markers of AD. Over the next 100 years, the disease has become increasingly frequent owing to increased life expectancy. Its neuropathological markers include the accumulation of beta-amyloid plaques and tau pathology. Currently, the amyloid cascade hypothesis is accepted, in which the first event of the disease is the deposition of amyloid-beta (Aβ), followed by synaptic loss, formation of neurofibrillary tangles and neuronal death (for a review, see [7,8]).

### 1.4. Alzheimer’s Disease Physiopathology

The pathophysiology of AD involves changes at the brain level, including the accumulation of two proteins: Aβ and hyperphosphorylated tau protein [43]. AD currently has no unequivocal premortem diagnosis and can only be diagnosed histologically postmortem in the presence of (1) the deposition of senile plaques, (2) neurofibrillary tangles and (3) neuronal death [7].

Senile plaques are characteristic alterations of AD, quite common in the brains of patients with dementia, and are possibly the origin of the denervation of the disease. Senile plaques result from progressive accumulation of parenchymal Aβ [7]. Aβ is a 39–43 amino acid peptide derived from progressive processing of the beta-amyloid precursor protein (APP) by β- and γ-secretase complexes, where presenilin (PS) is the catalytic component [44]. Aβ is derived from the proteolytic cleavage of the APP protein, which, when processed by β- and γ-secretases, results in three products, including Aβ, and favors the formation of senile plaques. In contrast, when α-secretase acts, APP is cleaved such that its products do not enter the amyloidogenic pathway [7]. Among the possible Aβ isoforms, Aβ40 and Aβ42 are the most common, with Aβ42 being the most fibrillogenic. Accumulation of Aβ aggregates as soluble oligomers and senile plaques plays a relevant role in the pathogenesis of AD [45]. The accumulation of senile plaques can cause interference with neuronal communication, triggering an inflammatory response in the brain, generating oxidative stress at the brain level, activating toxic processes that interfere with normal cellular processes, altering synaptic function and causing neuronal degeneration [43,46]. The pathology and dynamics of senile plaque formation and remodeling, as well as the precise involvement of amyloid deposition, are not fully understood, although senile plaques remain the main therapeutic targets in the development of new alternatives to prevent or reverse the disease [7,8,47]. Aβ peptides in their different aggregation states and compact senile plaques are neurotoxic both in AD and in experimental models [48] and have been associated with synaptic loss and the development of neuritic dystrophies [49,50,51]. Compact senile plaques have also been associated with abnormal curvature of nearby neurites [52,53,54] and may alter cortical synaptic integration [55,56]. In addition, senile plaques tend to accumulate in regions such as the cortex, hippocampus and cortical associative areas. This accumulation of plaques has a significant impact on cognitive functioning, including impairments in attention and concentration, language and communication difficulties, memory impairment and deficits in executive function [46].

### 1.5. Vascular Dementia Physiopathology

**Figure 3:**
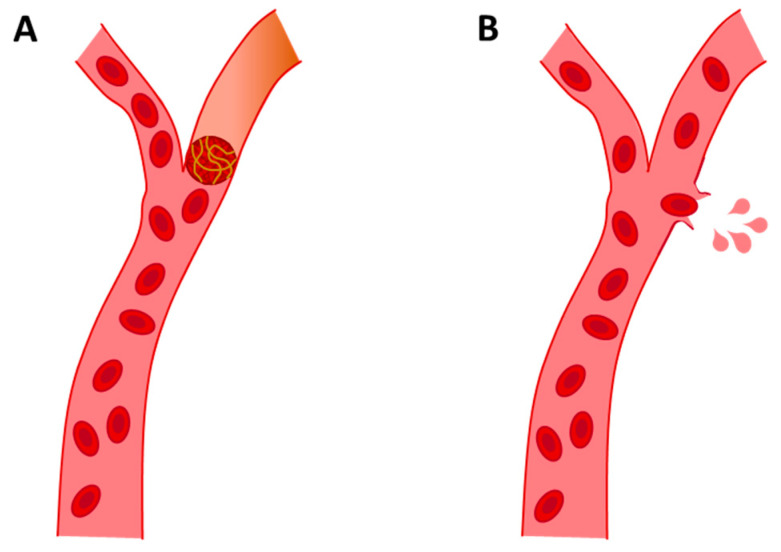
Representative images of ischemic stroke (**A**) and hemorrhagic stroke (**B**).

After AD, which is responsible for about 50–75% of all dementia cases [4], VaD is the second cause of dementia. VaD is a clinical cognitive disorder of cerebral vascular origin caused by stroke. The main cause of VaD is cerebrovascular disease, especially cerebrovascular accidents or strokes [57]. This leads to the degeneration of the affected area, usually the cortex, white matter or both, due to hypoxia. Oxygen deprivation preceded by hypoperfusion can be caused by different vascular aetiologies [58,59]. Broadly speaking, there are two types of strokes: (a) ischemic and (b) hemorrhagic. The main cause of VaD is ischemic stroke, which is caused by vascular collapse that impedes blood circulation, resulting in cerebral hypoperfusion. This deprivation of blood supply can be acute or progressive, depending on the degree of hypoperfusion [60]. VaD from hemorrhagic stroke is caused by vascular rupture, which results in blood contents spilling into the extracellular space (see Figure 3) [61]. Hemorrhagic strokes account for approximately 15% of all strokes, increasing the probability of dementia [60].

VaD is composed of a cumulative stroke process that encompasses a heterogeneous pathology of multiple microinfarcts, ischemic small vessel disease, microvascular damage [62] or even the deposition of Aβ in the form of amyloid angiopathy around the cerebral vessels [63]. This heterogeneity of VaD contributes to the presence of numerous clinical phenotypes causing difficulties and controversies in its diagnosis and classification [64,65,66,67].

The pattern of cognitive impairment observed in VaD is variable and can be difficult to distinguish from progressive cognitive decline, especially in the early stages when episodic memory is affected, and it can also be found in the early stages of AD [65]. However, whereas age-related cognitive decline is usually slower, in VaD, it occurs more abruptly and fluctuatingly [68]. Executive function is affected early in VaD to a greater degree than in other dementias, possibly due to the disruption of frontal connections [69]. Similar to AD, mood swings and personality alterations are very pronounced in VaD [67,70]. Another characteristic is decreased serotonin metabolism and a deficit of cholinergic markers, like that described in AD [71,72]. Lesions in white substances can directly affect cholinergic projections. Preclinical and clinical evidence suggests that the cholinergic system may also be involved in VaD [73,74,75]. On the other hand, cognitive impairment associated with subcortical vascular damage may be the result of cortical atrophy of the hippocampus, and although the cause of diffuse cortical atrophy is not well understood, it can be partially correlated with the severity of white matter lesions [75,76].

VaD and AD coexist, and it is possible that vascular pathology contributes to cognitive decline. Thus, it has been speculated that vascular damage may promote AD development. In this sense, blood–brain barrier (BBB) dysfunction could affect the Aβ brain–periphery balance and thus contribute to parenchymal and vascular Aβ deposition [77,78,79]. Furthermore, the pathology of AD can cause vascular damage, such as when Aβ deposition induces inflammation and endothelial damage. The pathological process resulting from vascular damage is associated with alterations in functional markers, such as increased oxidative stress, elevated proinflammatory cytokines or increased activity of matrix metalloproteinases in vessel walls [80,81]. These processes have been linked to neuronal death [82,83] responsible for dementia.

The ultimate triggering cause of VaD is not fully known, although a multitude of studies have been conducted on the relationship between metabolic disorders, lifestyle and vascular dementia [84,85]. In the 1990s, the link between DM and some dementias, such as AD and DaV, was already discussed [86]. The literature suggests that insulin resistance associated with T2D promotes cerebrovascular dysfunction, which would provide a favorable environment for the development of VaD and AD [84,85,87,88]. This review aimed to explore the underlying relationship between T2D, AD and VaD.

## 2. Link between Diabetes Mellitus, Alzheimer’s Disease and Vascular Dementia

Over the last 15 years, the relationship between T2D, AD and VaD has been extensively studied. Based on published studies, the data suggest that T2D, among other metabolic pathologies, could contribute to the development of a neurodegenerative process that would be a precursor to the development of dementia. Following this idea, DM seems to play a role at this level. The bibliography shows that the incidence of both pathologies increases with age, and it is common to find them coexisting. In fact, some authors claim that 45% of people with T2D suffer from mild cognitive impairment [89], raising the chance of developing dementia by 1.5–2.5 times [90]. In contrast, people with established dementia show insulin disturbances, with altered fasting glucose levels [91].

It also appears that the risk of dementia increases as patients develop other risk factors, including heart disease, hyperlipidemia, hypercholesterolemia or smoking, although T2D is important for its synergistic capacity [34,84,90]. In this relationship, insulin levels and insulin resistance are best correlated with the severity and progression of VaD [92,93]. Furthermore, it seems that DM promotes vascular damage caused by high levels of glucose in the blood, which is accentuated if it coexists with other pathologies that affect blood vessels, such as arteriosclerosis and hypertension [32,94].

The relationship between T2D and VaD can be based on processes that converge between the two pathologies, described below.

### 2.1. Insulin Receptors in Central Nervous System

Insulin receptors at the central level are located in astrocytes and neuronal synapses and are very abundant in regions of the basal forebrain, such as the septum (origin of cortical and hippocampal cholinergic innervation) [95], and areas particularly relevant for learning and memory processes, such as the cortex and the hippocampus [96]. This wide distribution of insulin receptors explains the involvement of insulin in cognitive processes [97], probably mediated by relevant neurotransmitters in AD, such as norepinephrine and acetylcholine [98,99]. Insulin also contributes to synaptic plasticity mediated by insulin receptors [100]. Similarly, insulin regulates glucose metabolism as the main nutrient of the CNS, directly involved in learning and memory processes [101,102]. In fact, the acute administration of insulin to humans and rodents produces an improvement in cognitive processes and memory [103,104,105,106,107] as well as an increase in the expression of insulin receptors in the dentate gyrus, leading to better performance in spatial memory tests [106,108]. Indeed, some authors have postulated that insulin could counterbalance AD pathology [109].

### 2.2. Type 2 Diabetes Progression Correlated with Pancreatic Amilin Deposition as Brain Aβ Deposition Correlated with Alzheimer’s Disease Progression

The progression of T2D is correlated with pancreatic amylin deposition, which is similar to that of brain Aβ. In addition, insulin, amylin and Aβ are degraded peripherally by the insulin-degrading enzyme (IDE) [110]. This suggests that these substrates may compete at this level [111]. It has been postulated that substrate imbalance may influence the pathogenesis of AD and T2D [112].

From another point of view, insulin resistance detected in AD patients could be mediated by a decrease in the activity of the enzymes responsible for its degradation. Both the insulin-degrading enzyme (IDE) and neprelisine (NEP) are involved in insulin degradation as well as Aβ and amylin degradation [113]. Accordingly, it has been postulated that an imbalance of substrates can affect the degradation rate of other substrates and possibly influence the pathogenesis of T2D and AD [112,114,115,116]. A decrease in IDE expression may result in reduced insulin and Aβ degradation in the brain [115,117]. Consequently, it is conceivable that actions promoted by elevated insulin levels, or its deficiency, may also be a link between T2D and AD.

### 2.3. Insulin-Like Growth Factor

Insulin and the insulin-like growth factor (IGF) have similar structures and play a significant role in the regulation of aging. IGF acts as a cellular growth factor but also plays a hormonal role in regulating growth and metabolism at the systemic level [118]. IGF is the main prenatal and postnatal growth factor. This is why low levels of insulin during gestation can lead to slower growth and low height and weight of the offspring [107,119]. In contrast, an increase in insulin levels at an early stage, as occurs in maternal T2D, leads to large and overweight children, among other complications [120].

In this context, insulin plays a crucial role in the development of neuronal complexity, as well as in neurogenesis in early neonatal development. In this sense, insulin deprivation during early life may result in reduced neural network development, whereas exogenous administration of insulin may promote increased neural development and complexity [107,121]. A more complex neural network is a protective factor against the development of dementia [122]. Based on the aforementioned, a gestational environment with altered insulin levels could condition the possible development of dementia in the later stages of life [123,124].

Additionally, plasma inulin can cross the BBB in a soluble form and gain access to neurons, microvasculature and even immature neuronal bodies [125]. Insulin plays a key role as a growth-regulating hormone during the early stages of life. In animal models, it has been shown that an imbalance in insulin levels also impairs neurogenesis in early life [107], as well as in more mature and long-lived phases [37]. In addition, the coexistence of T2D with AD pathology reduces neurogenesis and thus neuronal replacement from stages prior to cognitive decline [126]. Insulin is also involved in the regulation of neuronal and synaptic functions in the hippocampus, cortex and cerebellum, protecting neurons from neurodegeneration and cell death [127,128,129,130].

### 2.4. Insulin Promotes Typical Features of Alzheimer’s Disease

Tau pathology is one of the main features of AD, and many studies have reported the impact of insulin dysfunction and diabetes on it [131,132,133]. Indeed, high insulin levels in the brain, induced by prediabetes or T2D, may increase the hyperphosphorylation of tau protein [134]. It is feasible that higher levels of phosphorylated tau observed in hyperinsulinemia and T2D states could be mediated by insulin receptors at the central level [132]. Additionally, T1D hypoinsulinemia may increase tau hyperphosphorylation [135]. In this regard, a clinical phase I study with the drug SCR-1693 showed a reduction in tau protein phosphorylation levels associated with an improvement in cognitive and central insulin resistance [136].

Inflammation of the CNS is increasingly regarded to play a role in cognitive disorders such as dementia [137]. Insulin promotes the expression of proinflammatory cytokines such as α-TNF and IL6 [138], which are the most important proinflammatory cytokines. Moreover, these cytokines negatively affect the metabolism of Aβ oligomers [139]. Similarly, a proinflammatory state, in concomitance with amyloid pathology, leads to the activation of microglia and astrocytes in the SNC [140]. A recent study in several models of diabetes combined with a classical AD model, such as the APP/PS1 mouse, concludes from the cytokine profiles exhibited in each pathology that neuroinflammation may be the mechanism by which diabetes affects the pathology of AD [80].

On the other hand, one of the mechanisms proposed to link insulin to cognitive impairment is its role in Aβ metabolism [111,115]. In this regard, insulin promotes the amyloidogenic pathway by modulating β and γ-secretases [114,141,142]. Additionally, insulin inhibits or hinders the passage of Aβ through the BBB [143]. Following this idea, insulin would prevent the clearance of Aβ and promote its accumulation in the brain [144]. In return, Aβ interferes with insulin signaling in the CNS; in fact, soluble Aβ oligomers may disrupt insulin signaling in hippocampus neuronal cultures [145]. Furthermore, insulin promotes Aβ deposition in the brain in a similar way to amylin deposition in the pancreas of T2D patients. Amylin, like Aβ, is a toxic species involved in the apoptosis of pancreatic cells and neurons [146,147]. Moreover, amylin and Aβ aggregates alter cellular function by similar mechanisms: mitochondrial dysfunction and the formation of reactive oxygen species [148].

### 2.5. Role of Prediabetes and Diabetes Mechanism in the Neurodegenerative Process of Alzheimer’s Disease and Vascular Dementia

Previous epidemiological and clinical studies support a close relationship between T2D and AD [148,149]; however, the underlying linking mechanisms are not yet fully understood. It also remains unclear whether hyperinsulinemia and insulin resistance, indicative of a prediabetic state prior to T2D, may induce or accelerate central pathology in AD, in a similar manner to that induced by T2D. Indeed, glucose and insulin play a crucial role in maintaining normal brain activity, and alterations of insulin-dependent functions could be associated with central pathological conditions observed in AD [139,148,150].

The available scientific evidence on this relationship supports that the first pathological event in the DM disorder, as a promoter of dementia, is insulin resistance in the CNS [92,125,151]. Insulin plays an important role in cell growth and differentiation, as well as in protein synthesis [125,152]. Additionally, insulin inhibits catabolic processes such as glycolysis, lipolysis and proteolysis [152]. The wide distribution of insulin receptors throughout the CNS underscores its importance in central glucose homeostasis processes and its role in cognitive processes and neuronal development [125]. In this sense, it has been described that alterations in insulin balance in the CNS accelerate the brain aging process, increasing vascular damage and primarily affecting synaptic plasticity [50,152,153]. Vascular damage is often one of the first central events in diabetes [154], even in the prediabetic stages, and compensatory high insulin levels are sufficient to cause this damage [39]. Simultaneously with vascular damage, brain aging occurs, which is translated into reduced neuronal arborization and synaptic density. The loss of neuronal and synaptic density has been proposed as one of the best pathological markers for the assessment of AD. There is ample scientific evidence that different states of Aβ aggregation, from the most soluble forms to the formation of senile plaques, are neurotoxic [155,156,157]. It has been reported that synaptic loss promotes cell dedifferentiation and ultimately neuronal death [158]. Studies in animal models have shown that T2D conditions decrease synaptic density at the cortical level [38]. This decline is exacerbated when prediabetes and T2D coexist with AD, with greater involvement observed in regions close to senile plaques than in areas away from them [50]. One plausible explanation for this increased involvement is that diabetes, by some yet unknown mechanism, may be promoting soluble and more toxic forms of Aβ [78,79], which would allow for a more extensive distribution and involvement of the brain [159]. However, this effect has been observed in both T1D and T2D animal models [78,79]. The overproduction and absence of insulin produced in T2D and T1D, respectively, lead to vascular damage and a chronic proinflammatory state [38,80]. T1D promotes vascular changes, such as impaired regulation of vascular tone and dysfunction in neovascularization and vasoregression, leading to a blood–brain barrier disruption (for a review, see [160]). T2D can also promote vascular dysfunction and inflammation (for a review, see [161]). Both factors may contribute to a lower capacity to eliminate excess Aβ vascularly or through microglia. This situation promotes its accumulation in a soluble form. In fact, in our murine models of AD-T2D and AD-T1D, the levels of serum Aβ were reduced, indicating the inability to expel peptides from the CNS across the blood–brain barrier, while intracerebral soluble Aβ levels were increased [78,79]. In fact, the higher levels of soluble Aβ40 and 42 in the CNS observed in T1D and T2D are also in agreement with large synaptic loss and neuronal death observations [79,81,162,163,164,165,166]. It is feasible that the shift toward more toxic soluble species might contribute to the observed progressive synapse reduction in prediabetic and diabetic AD mice [50,167] and an in silico study [168]. In addition, prediabetes and T2D promote an increased accumulation of Aβ in the form of amyloid angiopathy (AAC) around blood vessels [81], which is associated with an increased risk of vascular rupture [169]. On the other hand, Aβ clearance might also be affected. In this regard, some studies support that Aβ pathology, in combination with hyperinsulinemia, promotes higher levels of Aβ because IDE is much more selective for insulin than for Aβ [115,170]. In addition, the lack of insulin in T1D seems to be associated with lower levels of enzymatic activity (for a review, see [171]). Additionally, previous studies have shown that the activity of IDE and NEP could be intrinsically altered in AD or DM [163,172,173]. Likewise, vascular damage observed in prediabetic-AD and T2D-AD mice could affect Aβ clearance by altering the BBB, in accordance with previous models showing reduced amyloid clearance along interstitial fluid drainage pathways [174,175], by damage of the artery feeding a particular brain territory.

In the second stage, we propose that the sequence of events involves the inflammatory process and glial activation. It has been suggested that the insulin resistance typical of prediabetes and T2D may exacerbate the inflammatory process when it interacts with the presence of Aβ [139]. Previous studies report that the presence of Aβ is sufficient to promote microglial activation and the inflammatory process [176,177]. Vascular damage and the T2D-induced imbalance of Aβ pathology toward more soluble forms have been shown to induce an increase in cytotoxic and pro-inflammatory cytokines and increase microglial activity [80,178]. This inflammatory process would increase oxidative molecular species, generating a harmful environment that would promote neurodegenerative processes [179]. Microglial cells, faced with vascular damage and the accumulation of toxic substances such as Aβ, begin to produce proinflammatory cytokines (IL-1β, IL-6, IL-18 and tumor necrosis factor-α (TNF-α)), chemokines (CCL1, CCL5, CXCL1), small messenger molecules (prostaglandins, NO) and ROS [180]. Although most cells involved in this process of neuroinflammation are microglia and astrocytes, capillary endothelial cells are also involved, as well as some infiltrating blood cells, which are more frequent when there is tissue damage in the BBB [180,181]. This pro-inflammatory ecosystem could lead to synaptic dysfunction, neuronal death and inhibition of neurogenesis [80,126,182]. Similarly, it has been shown that the increase in prostaglandin 2 by the damaged vascular endothelium increases the production of IL-1β, which is associated with synaptic loss through the activation of the postsynaptic N-methyl-D-aspartate receptor [182]. Under these circumstances, activation of the complement system, which promotes phagocytosis by microglia, may also be involved in the process of synaptic loss [183]. The described mechanism involves neuronal death by TNF-α-mediated caspase-8 recruitment and TNF receptor type 1 [184].

The following event would affect one of the classic AD pathologies: the hyperphosphorylation of the tau protein [7], which can also be found in other neurodegenerative diseases such as VaD. Previous studies have shown early tau phosphorylation in diabetic mouse models [185], whereas other studies support the idea that this pathology only appears in the latest phases of diabetes [38]. This apparent discrepancy may be explained by the fact that tau phosphorylation is highly dependent on the specific phosphorylated residues studied [185,186]. Altogether, these data support the step-through role of early prediabetes and consolidated T1D and T2D at the central level, which seem to preferentially affect the cortex and spread to the hippocampus as the disease progresses [39,78,79,187,188,189].

Described central alterations ultimately lead to learning and memory dysfunction. Prediabetes or early diabetes does not affect learning or memory [38,39]. However, late diabetes is related to poor cognitive preservation, and episodic and spatial memories are affected in animal models [38]. A similar outcome was observed in patients with T2D [190,191] and T1D [192], with lower levels of global cognition and episodic or working memory. In fact, a recent study supports that low levels of cognition in aging people are associated with body fat and higher body max index, which are risk factors for suffering AD, VaD and T2D [193]. Metabolic parameters are correlated with CNS alterations in animal models; a study supports that glucose levels, insulin levels and body weight are good predictors of cortical atrophy and impaired cortical and hippocampal cell proliferation, suggesting the role of the diabetic process at the central level [37]. In accordance with this, human studies have also shown significant associations between insulin levels in T2D patients and brain alterations detected by magnetic resonance imaging (MRI) [189,194,195]. Curiously, whereas worse metabolic conditions correlate with lower rates of central cell proliferation, affected metabolism also predicts increased neurogenesis rates, suggesting that as the pathology progresses, the overall cellular production is impaired, and the system tries to compensate for this by generating new neurons [37].

## 3. Future Perspectives

Growing evidence suggests that T2D may increase the risk of developing AD. Several studies have found that insulin resistance and metabolic dysfunction associated with T2D negatively affect the brain and increase the accumulation of Aβ. Also, T2D promotes chronic inflammation, oxidative stress and vascular dysfunction in the brain which links T2D with AD and VaD.

Understanding the relationship between DM and AD is essential to prevent the development of AD through metabolic control. Likewise, knowledge of the involvement of DM in the progress and development of AD can help us address new therapeutic strategies for its treatment. In this sense, treatment to control T2D, such as metformin, has shown a beneficial effect, like neuroprotective, anti-inflammatory and antioxidant action in animal models [196,197,198]. However, limited studies in humans have shown a more discrete positive relationship with the use of metformin as a treatment for AD [199]. The possible beneficial effects of metformin as a treatment for Alzheimer’s disease are still being studied and are a hot topic (for a review, see [200]).

Another emerging candidate for a therapeutic approach to AD an VaD is the application of intranasal insulin. This treatment has been shown to have beneficial effects in reducing inflammation and improving immune function. In addition, intranasal insulin improves cognitive status and helps to maintain cognitive abilities [201,202]. Other studies have shown that intranasal insulin as a treatment for AD has neuroprotective effects, and its use can maintain the insulin brain signaling to improve cognitive health [203] by reducing the P-tau/Aβ42 ratio [204] and preserving the white matter volume in deep and frontal regions, which correlates with AD progression [205]. However, recent long-term studies in humans have shown limited efficacy of intranasal insulin as a treatment for AD [204,206]. The current disagreement in the literature shows the need to carry out studies that demonstrate the usefulness of this treatment in the control of AD.

Finally, as the understanding of the relationship between T2D, AD and VaD increases, there are new opportunities for prevention and treatment. Improving glycemic control and promoting healthy lifestyles should be explored further. Thus, early attention to risk factors associated with dementia, such as T2D, may play a crucial role in preventing or delaying dementia.

## 4. Conclusions

**Figure 4:**
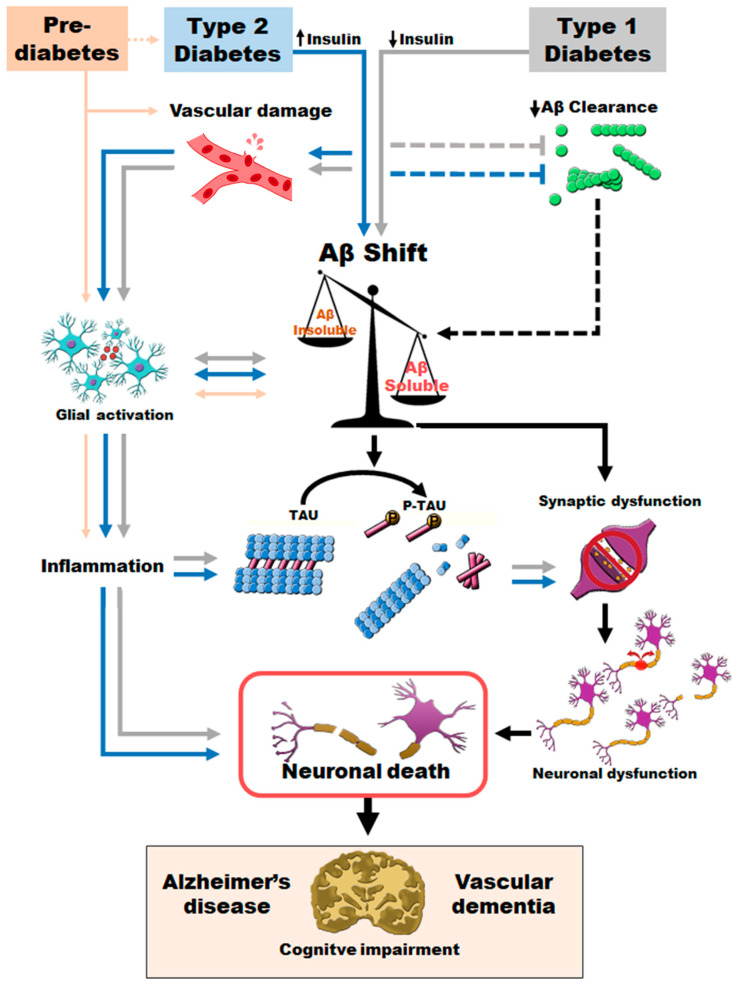
Proposed sequence of pathogenic events promoted when T2D or T1D coexist with AD and VaD.

The findings of this review clarify the underlying relationship between AD, T2D and VaD. Thus, early hyperinsulinemia, without clinically established T2D, is sufficient to worsen AD pathology. T2D promotes vascular damage and increases inflammatory processes at the central level. Moreover, both T2D and T1D accelerate AD pathological features, increasing more toxic Aβ species and resulting in a more severe version of AD (Figure 4). Increased levels of soluble Aβ and phosphotau may contribute to synaptic loss, neuronal death, brain atrophy and final cognitive impairment. Altogether, T2D promotes an early and more severe version of AD and VaD pathology. Published evidence provides a relevant tool to further explore the relationship between T2D, AD and vascular implications, offering the possibility to assess therapeutic approaches that can delay or prevent AD and VaD pathology by improving metabolic control.
